# The correlation of functional pain and psychological distress: a study in Italian school students

**DOI:** 10.1186/s13052-019-0668-0

**Published:** 2019-07-12

**Authors:** Margherita Lo Curto, Maria Cristina Maggio, Fabio Campisi, Giovanni Corsello

**Affiliations:** 1 School of Specialisation in Paediatrics, University of Palermo, Palermo, Italy; 2 Department of Health Promotion Sciences Maternal and Infantile Care, Internal Medicine and Medical Specialities “G. D’Alessandro”, University of Palermo, Palermo, Italy; 3 Ospedale dei Bambini G. Di Cristina, via Benedettini 1, 90143 Palermo, Italy

**Keywords:** Functional pain, Psychological disagreements, School fellows’ relation

## Abstract

**Background:**

Functional Pain (not detectable organic cause) is often associated with psychological problems and, according to literature, it can lead to severe manifestations.

The purpose of the study was to investigate the correlation between functional pain and psychological disagreement, in a series of school students.

**Methods:**

An observational questionnaire-based study was performed.

A questionnaire was given to a group of students of primary school; the following data were collected in the questionnaire: a) sex and age; b) functional pain; c) relation with relatives, teachers and schoolfellows: d) school failure.

Statistical methods: *P*-value of concordance test and *P*-value of correlation have been performed with MINITAB 15.1 software.

**Results:**

Eight hundred nine students, 354 females, 455 males, median age 14 years, participated to the study.

Functional Pain was referred from 537/809 students (66%): 265 Females, 272 males: *p* = 0.155. The difference between the number of pain episodes in females vs. males was statistically significant (*p* = 0,511), as pain intensity vs. the number of episodes in females (*p* = 0.001).

The most frequent location of pain was abdomen in females, limbs in males.

Psychological disagreement was referred from 513/809 students (63%) (260 females; 253 males: *p* = 0,150).

Psychological disagreement was reported with parents (19); siblings (22); other relatives (18); teachers: 42, schoolfellows: 366, relatives as well as school fellows: 46.

The correlation between disagreement and functional pain in all the students included in the study was statistically significant (*p* < 0.001).

**Conclusions:**

most students reported psychological disagreement and pain. The most frequent cause of disagreement was schoolfellows’ behaviour. The study shows a student’s lack of discussing of their problems with parents, teachers, peer. According to literature, confiance would be a useful treatment for avoiding psychological disagreements and functional pain.

## Introduction

Psychological distress occurs frequently in children and adolescents; moreover, some of these suffer from pain, whose organic, metabolic or traumatic cause is not demonstrable. Such pain is defined as “functional pain”; it is common in children, who suffer of psychological diseases, often accompanied by stress and traumatic life events [1–6]. The relationship between anxiety depression and pain is complex; large majority of these children and adolescents, have no serious or treatable physical cause [4, 7–9]. Functional pain is more frequent in females than in males [10], and the most common sites are head and abdomen.

A longitudinal study showed that 50% of students referred head pain, abdominal pain, or pain in musculoskeletal tissues; the pain decreased with age in boys and increased in girls [8]. In a study performed on Dutch adolescents, Diepenmaat [4] reported (particularly in girls) the prevalence of neck/shoulder and low back pain lasting for ≥4 days per month; pain was associated with depression and stress**.**

Negative effects of functional pain frequently occur. Functional abdominal pain is frequently associated with other functional gastrointestinal disorders caused by psychological distress [11].

Impaired quality of life as emotional, social, school disagreements, is frequent, in young with functional pain, as showed in a study which included 313 girls and 292 boys (mean age: 9.7 years) and 386 girls and 464 boys (mean age: 12.6 years) suffering of functional pain; the majority of children with recurrent pain, referred unpleasant quality of life [12] and impaired functioning [13]; a somatoform disorder is reported in children who could not adapt to pain [14–16]; moreover, these children may present strong anxiety, and sensitivity, which are significantly catastrophizing feeling [13, 15, 16].

We emphasize that suffering of depression and/or anxiety are the causes at the origin of functional pain [1–8] and of bullying in both victims and actors [17, 18]. Fales [19] showed the correlation of peer victimization of bullying in adolescents with chronic pain.

The causes of disagreements in young people are complex: the relationship with parents, within the school and with their peers were investigated.

Parents distress is often the cause of children distress and of functional pain; furthermore, it has a role in the context of children’s pain treatment [20–23].

Cunnigam et Al [23], showed that the psychological feeling of children is related to the presence of significant anxiety in parents; furthermore, parents anxiety contributes to school-related functional impairment [24].

School anxiety is a feeling among youth with chronic pain. The way in which school anxiety is experienced in paediatric chronic pain is a problem to be investigated [25]. Conversely an authoritative school climate characterized by strict but fair discipline and supportive teacher-student relationships is useful to reduce negative feeling in adolescents [26].

Children are unlikely to be comforted is reasonable to suggest further studies concerning unexplained children’s pain and care to alleviate their psychological and emotional suffering. For the above reasons, it appears mandatory to treat the functional pain, although the choice of treatment is controversial. Dietary, pharmacological, psychological treatments have been tried. For children with abdominal pain were reported:

i) data of treatment with cyproheptadine [27].
ii) carbohidrate restriction and soluble fibre supplementation [28];
iii) administration of *Lactobacillus reuteri* [29] that reduced the intensity of abdominal pain.

Improvement of gastrointestinal pain with placebo treatment has been observed; the Authors suggested eventual new studies concerning the role of placebo in functional pain [30].

A study by Cochrane [6] showed that, for recurrent abdominal pain, there is no evidence for the efficacy of diet modification as fibre supplements, lactose free diet, or lactobacillus supplementation. Psychological treatment, including cognitive and behavioural treatment have been used with statistically significant improvement of pain.

Antidepressant therapy has a significant efficacy [31]. The study of Eccleston et Al. [32] reported that frequently adolescents feel to be less clever than their peer; likewise, the support of the peer is useful for their psychological feeling, friendly relation and social development.

Self-efficiency can be a protective factor for psychological disagreements [33].

The Cochrane study [34] reviewed 37 studies concerning treatment of functional pain with the total number of 1938 participants, and concluded that, psychological therapies are efficacious for pain intensity reduction; the efficacy being long lasting.

Laurseen [35] suggests that cultural ethics has implications in the treatment of young people with functional pain.

In a previous study [3], concerning a group of 246 school students and a group of 194 children, hospitalized for painless diseases, we studied the psychological feeling and the occurrence of functional pain. This study showed that in most of the hospitalized patients as well in most students, the functional pain was associated with psychological distress. The main object of psychological disagreements, in both hospitalized and school students, were the relations with their schoolfellows; a small percentage referred disagreements with parents and/or relatives; however, in both groups, the correlation of psychological distress and functional pain, was not statistically significant.

We concluded that the lack of correlation of functional pain and psychological disagreements, of our reported series, required to study a wider number of cases.

In the present study, concerning a wide series of students, our objectives were to verify: i) the statistical significance of the correlation between psychological disagreements and functional pain; ii) the most frequent cause of disagreements referred by the students; iii) the percentage of students with functional pain, the site of pain, its intensity and the number of pain episodes, according to sex or age; IV); the benefice of pharmacological and/or dietary treatment adopted; V) if the students discuss of their psychological problems: with their parents, teachers, schoolfellows.

## Material and methods

The investigation was performed through a questionnaire concerning functional pain and psychological disagreements in adolescents.

The study concerns students of one lower secondary school and of three upper secondary schools, observed between January 2011 and December 2015.

The study included 150 students of 4 classrooms of lower secondary school, and 659 students of 31 classrooms of upper secondary schools.

Overall 809 students were investigated. For each class, the students were gathered in a meeting room at school. Two of the investigators described to the students the aim and the methodology of the study in the presence of one of their teachers and of the school psychologist.

Informed consent was obtained by the students and by their parents.

The study was approved by the Directors of the schools, and by the Ethic Committee (Chair Person: Professor Salvatore Leone).

A questionnaire was given to each student and the following data were required:

- sex and age;
- ; the relationship with parents, brothers/sisters, other relatives, schoolfellows and teachers;
- the pleasure in attending school;
- occurrence of pain not caused by organic, metabolic or traumatic events in the latest 3 months; location and intensity of pain, number of pain episodes;
- treatment of pain;
- occurrence of discussions of their problems with their peers, parents, teachers.

For the sake of privacy, students signed their questionnaires with a fantasy name.

**Exclusion criteria**: i) students suffering from any organic or metabolic disease, mental disability or psychiatric disease; ii) students assuming antalgic or psycho-drugs; iii) students with recent organic injuries.

**Inclusion criteria**: heathy students (age: 10–17 years) speaking Italian.

All the enrolled students agreed to participate to the study.

The forms were filled in 1 h and the data were collected by the investigators.

Pain intensity was evaluated by VAS scale (scores 0–10); high intensity score: 10–8; median intensity score: 5–7; low intensity score. 1–4.

### Statistical analysis

Statistical analysis, in terms of descriptive statistics, *P*-Value of concordance test and *P*-Value of correlation, has been performed with MINITAB 15.1 software. Logistic regression tool available in the software was adopted to investigate the relationship between a response variable and one or more predictors when ordinal and/or nominal categories were of interest.

## Results

The study included 809 students, 354 females, 455 males (age: 10–17 years, median: 14 years).

All the students were Italian, of the social middle class.

All the students who presented the required inclusion criteria, were potentially eligible and were included in the study.

537/809 (66%) reported pain and affirmed, in the questionnaire, that previous appropriate clinical, laboratory or imaging investigations suggested that their pain was not due to any organic, metabolic or traumatic events; accordingly, their pain can be defined as “functional”.

Pain was reported by 272 out of 354 (77%) females and by 265 out of 455 (58%) males.

The difference of the number of females and males who referred pain was not statistically significant (*p* = 0.155).

According to VAS scale, 12% of the students reported high pain intensity; 78% median pain intensity; 10% low pain intensity.

The number of episodes ranged from one to thirty: a single episode was referred by 22 students; five ÷ eight by 280 students; while 507 students did not remember the number of episodes. The duration of episodes ranged from 10 min to 4 h in 90% of cases and it was > 24 h in 10% of them.

Pearson correlation of number of pain episodes in females versus males was not statistically significant (*p* = 0.511).

Pain intensity showed statistically significant inverse correlation versus the number of pain episodes in females (*p* < 0.001); the correlation did not reach the statistical significance in males (*p* = 0.716).

The location of pain, according to sex, is reported in Table 1.

**Table 1:** Pain location, according to students’ sex

	Abdomen	Head	Limbs	Back	Total
Females*	114 (41%)	87 (31%)	68 (24%)	10 (4%)	279
Males*	66 (23%)	77 (26%)	138 (47%)	10 (3%)	291
Total	180 (32%)	164 (29%)	206 (36%)	20 (4%)	570

The abdomen was the more frequent site of pain for females, the limbs for males *28 females and 10 males referred more than one pain site

Twenty-nine students referred that their episodes of pain have been treated with analgesics without efficacy.

### Disagreements

- Out of 809 students, 513 (63%) reported psychological disagreements; 296 (37%) students did not report any disagreement.
- Psychological disagreements were reported by 260 out of 354 females (75%) and 253 out of 455 males (56%).
- Pearson correlation of disagreements in males vs. females was not statistically significant (*p* = 0.150).
- 19 students reported disagreements, as it is shown in Table 2.
- 366 students who had bad relationships with schoolfellows, referred that their disagreement was mainly related to the behaviour of their fellows. The students disliked “how some of them treated their peers”.
- 46 students reported disagreements with relatives as well as with school fellows.
- In only two cases displacement in attending school was referred.
- No talking of their personal problems with their peers, parents, teachers or other relatives was referred.
- Neither difference of pain nor of disagreements related to the age of the students, was deducible.

**Table 2:** Disagreements of the students

	Students with disagreements
Parents	19 (3.7%)
Brothers or sisters	22 (4.3%)
Other relatives	18 (3.5%)
Teachers	42 (8.2%)
School Fellows	366 (71%)
Relatives and school fellows	46 (9%)
Total	513

As showed in Table 3, in all the cases we studied, disagreements were significantly correlated with reported pain (*p* < 0.001).

**Table 3:** Correlation of disagreements vs. pain in 809 students

	Students	Pain	No pain
Disagreements	513 (63%)	404 (50%)	109 (13%)
No disagreements	296 (37%)	133 (16%)	163 (20%)
Total	809	537	272

Data show that functional pain was present more frequently in students who reported psychological disagreements (404: 50%); only a few not suffering psychological disagreements referred functional pain (133: 16%)

The correlation “disagreements vs. functional pain” was statistically significant (*p* = 0.001)

No missing data were observed in the reported questionnaire

## Discussion

As above mentioned, our previous study, concerning a series of healthy school students [3], failed to show a statistically significant correlation among functional pain and psychological disagreements; here we attempted to detect such correlation.

Many of the students, in this, as in the previous study, reported psychological disagreements, as well as pain; the absence of organic, traumatic, metabolic lesions leads to define their pain as “functional”.

The study demonstrates, with a statistical significance, the correlation of functional pain with psychological disagreements, suggesting that disagreements are the main cause of functional pain and of its maintenance and recurrence.

In the present series, the pain was more frequent in females than in males, as reported in our and in studies by other authors [1–4, 10, 12]; however, the difference of reported pain, in females vs. males, was not statistically significant.

It is interesting that in girls who reported a high number of pain episodes, the intensity of pain was significantly lower than in those who reported only a few episodes: is it possible that girls who had several episodes of pain, did not consider the pain intensity?

The prevalent locations of pain were the limbs in males, the head and the abdomen in females. Headache is frequent in adolescents, mainly in females, as reported [8], neck/shoulder and low back pain, referred in the study of Diepnam [4] was attributed by the too frequent use of computer; this question was not asked to the students of our series.

Concerning the causes of disagreements, only a few students referred problems with teachers or study failure.

As above referred, the relationship of students with their parents may affect the psychological feeling of young adolescents.

Only a minority, as in the previous study [3], reported disagreements with their parents or other relatives.

As above referred, catastrophizing emotions of parents are associated with children anxiety, this might be the cause of functional pain [20–23]. We did not investigate the kind of feeling or the quality of life of the parents; that is why we do not know whether the parents’ feeling influenced the disagreements of our students.

It must be remarked that most of our students do not talk of their problems, neither with parents, or teachers or peer.

Several parents investigated in our previous study, gave answers different from their children to the question concerning the psychological distress and functional pain [3]. As we already reported [3], parents often underestimate the reality of the pain and the problems of the school relationships, we believe that the kind of relation of the students with their parents is a relevant limit of this study.

It would be in order to perform an investigation concerning the parents’ psychological behaviour and the concordance with the feeling of their children.

Most of our students did not refer school intolerance, or study failure.

The most frequent cause of disagreement of the students included in our study, was rejection by students of their schoolfellows’ behaviour, mainly concerning the kind of relation of some of them with some other school fellows. This reveals lack of friendship and of confidence.

Our study shows that the main problem for students is a lack of confidence in their teachers, in parents and other relatives, or in somebody disposable to listen them; moreover, it results that these adolescents were disappointed by the behaviour of their peers.

The above-mentioned correlation of parent’s behaviour and psychological distress in adolescents, leads to suggests that it would be a benefit to address parents to the confidence in their children, in collaboration with school teachers and, in some cases, with psychologists. It must be stressed that for children and adolescents are relevant the relationships with their peers.

In a minority of cases (in the present as in the previous study [3], functional pain was not related to psychological disagreements; we believe that other factors, such as family, social, ethnic, education factors or minimal organic lesions might influence the cortical centres of pain, and causes functional pain [36–38].

It must be noted that functional pain is a multifactorial condition that results from a complex interaction between psychosocial and physiologic factors via the brain-gut axis [38], that in some cases is not possible to rationalize.

## Conclusions

The reported data indicate that: I) most students referred functional pain and psychological disagreements; II) they disapprove their peers’ behaviour, being this, in our series, the major cause of psychological disagreement; III) the correlation between psychological disagreements and functional pain is statistically significant; IV) there is a lack of confiance of students in their parents, teachers and peers.

According to the literature, the analysis of our data suggests that it would be useful to suggest parents, teachers and peers to discuss the problems with adolescents; a better treatment of functional pain, would be to reassure them for gaining their confidence.

## Data Availability

The datasets used and analysed during the current study are available from the corresponding author on reasonable request.
